# Effects of cellular iron deficiency on the formation of vascular endothelial growth factor and angiogenesis. Iron deficiency and angiogenesis

**DOI:** 10.1186/1475-2867-10-28

**Published:** 2010-08-19

**Authors:** Jonathan Eckard, Jisen Dai, Jing Wu, Jinlong Jian, Qing Yang, Haobin Chen, Max Costa, Krystyna Frenkel, Xi Huang

**Affiliations:** 1 Department of Environmental Medicine and New York University (NYU) Cancer Institute, NYU School of Medicine, New York, NY 10016, USA

## Abstract

**Background:**

Young women diagnosed with breast cancer are known to have a higher mortality rate from the disease than older patients. Specific risk factors leading to this poorer outcome have not been identified. In the present study, we hypothesized that iron deficiency, a common ailment in young women, contributes to the poor outcome by promoting the hypoxia inducible factor-1α (HIF-1α and vascular endothelial growth factor (VEGF) formation. This hypothesis was tested in an *in vitro *cell culture model system.

**Results:**

Human breast cancer MDA-MB-231 cells were transfected with transferrin receptor-1 (TfR1) shRNA to constitutively impair iron uptake. Cellular iron status was determined by a set of iron proteins and angiogenesis was evaluated by levels of VEGF in cells as well as by a mouse xenograft model. Significant decreases in ferritin with concomitant increases in VEGF were observed in TfR1 knockdown MDA-MB-231 cells when compared to the parental cells. TfR1 shRNA transfectants also evoked a stronger angiogenic response after the cells were injected subcutaneously into nude mice. The molecular mechanism appears that cellular iron deficiency elevates VEGF formation by stabilizing HIF-1α. This mechanism is also true in human breast cancer MCF-7 and liver cancer HepG2 cells.

**Conclusions:**

Cellular iron deficiency increased HIF-1α, VEGF, and angiogenesis, suggesting that systemic iron deficiency might play an important part in the tumor angiogenesis and recurrence in this young age group of breast cancer patients.

## Background

Young women (< 45 years old) diagnosed with breast cancer are well known to have a higher risk of dying from the disease than older patients because of greater early recurrence rates, increased aggressiveness, and mostly estrogen receptor (ER), progesterone receptor (PR) and human epidermal growth factor receptor 2 (Her2) negative or triple negative breast tumors [1,2]. The specific risk factors contributing to this poorer outcome in young patients have not been identified. Although a decline in total breast cancer cases was recently reported, a plot of age-specific breast cancer rates shows a decrease only among women >45 years old [3]. This dilemma calls for new research on identification of additional risk factors in this young group of patients.

Using data initially collected from the New York University Women's Health Study, we observed that serum iron and ferritin levels are significantly lower in pre- than in postmenopausal women [4]. A typical characteristic of young pre-menopausal women is the presence of menstrual cycle. Through complex interactions of hypothalamic-pituitary-ovarian (HPO) axis, the pituitary gland stimulates the ovaries to mature and release an egg every month [5,6]. When the egg is not fertilized, the endometrial matrix prepared for egg fertilization is shed in the form of blood. Because of this blood loss, young females have higher dietary requirements for iron than males and older females to prevent iron deficiency [7]. The final stage of iron deficiency is iron deficiency anemia (IDA). IDA has been known to the medical profession for several centuries as the condition in which a person has inadequate amounts of iron to meet body's demands [8]. In developing countries, about 50% of all women of childbearing age have IDA and, iron deficiency is also common in young women of industrialized countries, particularly in poorer, less educated, and minority populations [9,10]. Clearly, iron deficiency is a relevant health issue in young women, but its relationship to poor breast cancer outcomes has not been investigated to date.

Iron is an essential metal for all living organisms participating in cellular processes, such as DNA synthesis, enzyme functions, and oxygen transport. Cellular iron metabolism is homologous among most cell types; cellular iron homeostasis is primarily mediated by transferrin (Tf), transferrin receptor-1 (TfR1), and ferritin [11-13]. Tf is an iron transport protein with two iron-binding sites and is mainly found in the bloodstream, from which it circulates and delivers iron throughout the body. TfR1 is a ubiquitous membrane protein forming a complex with Tf, which initiates membrane endocytosis and serves as a major cellular iron uptake pathway. Excess iron within cells is stored in ferritin. When cells are iron deficient, iron regulatory proteins (IRPs) bind to iron responsive elements (IRE) in the 3' untranslated region (UTR) of TfR1 mRNA as well as 5' UTR of ferritin mRNA. The IRP binding to 3'-UTR results in TfR1 upregulation and increased iron uptake, but its binding to 5'-UTR of ferritin leads to its downregulation and decreased iron storage [12,14].

Iron depletion in cells by iron chelator desferoxamine mesylate (DFO) has been shown to increase hypoxia-inducible factor-1alpha (HIF-1α) [15,16]. HIF-1α is a critical transcription factor for the regulation of vascular endothelial growth factor (VEGF), a potent inducer of tumor angiogenesis and metastasis [17]. However, direct evidence of iron deficiency on VEGF is lacking and the effect of iron deficiency on tumor angiogenesis has not yet been studied. We have hypothesized that, in young premenopausal women, an iron deficiency caused by menstruation stabilizes HIF-1α, which increases the formation of VEGF. This mechanism results in premenopausal women being more susceptible to angiogenesis and, consequently, leads to a high recurrence of breast cancer [18]. To provide proof of the concept that iron deficiency plays a role in angiogenesis, we used shRNA techniques in the present study to knockdown TfR1 levels in the triple negative breast cancer MDA-MB-231 cells. Because TfR1 is the gatekeeper of iron uptake, knocking down TfR1 is similar to closing the door for iron entry; it physically disables iron uptake and causes stable iron deficiency in cells, thus mimicking IDA. Our results showed that iron deficiency in cells increased the angiogenic potential of the cells.

## Results

### TfR1 shRNA knockdown causing cellular iron deficiency and decreasing cellular responses to iron modulation

**Figure 1:**
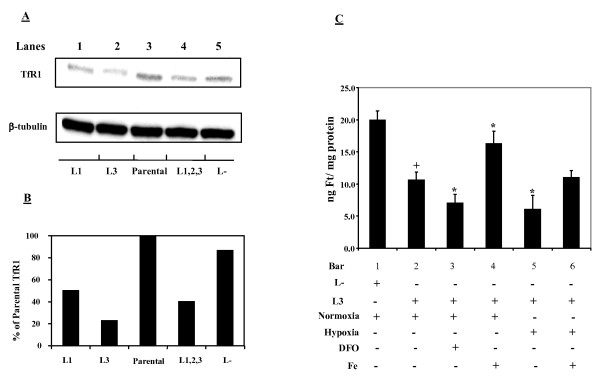
**TfR1 knockdown causes iron deficiency and decreases responses to iron modulations in MDA-MB-231 cells**. A: TfR1 levels in the parental MDA-MB-231 cells and TfR1-silenced transfectants. Lanes 1-2 represent stable transfectants, while lanes 4-5 illustrate TfR1 levels in cells collected after 24-h transient transfection. Lane 1: shRNA#1 (L1); lane 2: shRNA #3 (L3); lane 3: parental MDA-MB-231cells (control); lane 4: a mixture of shRNAs # 1, 2, & 3 (L1,2,3); lane 5: nonsense shRNA control (L-). B: Means of two densitometric analyses based on two independent transfection experiments. C: nonsense (L-) and TfR1 knockdown (L3) MDA-MB-231 cells were subjected to different treatments: DFO (0.5 mM; 6 h); hypoxia (1% O_2_, 6 h); iron (50 μM FAC, 6 h), and ferritin was quantitated by ELISA. Bars represent means ± SD of three independent experiments (n = 3). ^+^Significantly different from L- nonsense controls (p < 0.05). * Significantly different from L3 normoxic controls (p < 0.05).

Cellular iron deficiency was achieved by either transiently or stably transfecting MDA-MB-231 cells with TfR1 shRNA expression vectors of L1, L2, L3, or their mixtures (*e.g.*, L1, 2, 3, a mixture of the three oligonucleotides). Effective TfR1 knockdowns as confirmed by Western blot were shown in Figure 1A and the degrees of knockdown relative to the parental and nonsense control cells were shown in Figure 1B. It can be seen that cells stably-transfected with the L3 shRNA vector produced an 80% knockdown in TfR1 and this transfectant was selected for further studies.

Figure 1C shows that ferritin levels were decreased by 50% in the L3 TfR1 knockdown cells as compared to the nonsense control cells. When compared to the parental cells with baseline levels of ferritin at 22.1+1.5 ng/mg, a knockdown of 65% was observed. Iron chelation by DFO or hypoxic exposure further lowered ferritin levels in the L3 TfR1 knockdown cells (bars 3&5). As expected, iron supplementation caused elevation in ferritin levels in the L3 cells grown under either normoxia or hypoxia (bars 4&6). However, ferritin levels in the L3 cells treated with iron were still significantly lower than the background levels of ferritin in the nonsense transfected MDA-MB-231 cells without iron treatment. These results indicate that the L3 TfR1 knockdown cells are under iron-deficient conditions and are less responsive to iron modulation either by chelation or by supplementation.

### TfR1 knockdown cells enhancing VEGF production and *in vivo *angiogenesis

**Figure 2:**
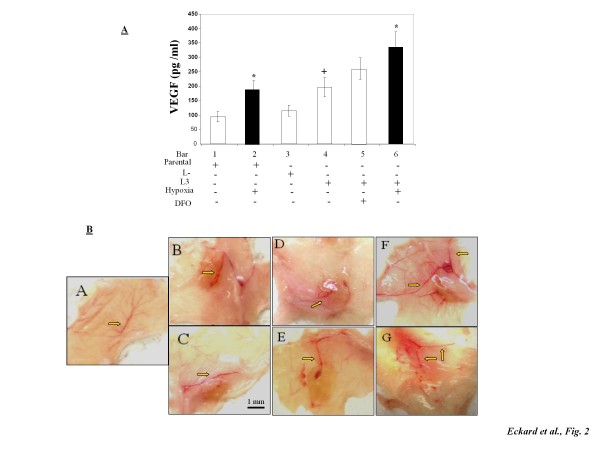
**VEGF production and *in vivo *angiogenesis by parental, nonsense, and TfR1 knockdown MDA-MB-231 cells**. (A) Levels of VEGF in tissue culture media of parental, nonsense (L-), and TfR1 knockdown (L3) cells treated with hypoxia or DFO. Bars represent the means + SD from four independent experiments (n = 4). (B) Matrigel plug xenografts (grown from MDA-MB-231 cells inoculated with Martigel™ into nude mice) and surrounding tissue were excised from the flanks after 72 h. Tissues were visually examined for the degree of vasculature recruitment to the xenograft site and arrows indicate neovascularization penetrating into the xenograft. Panel A: non-injected tissue; Panels B and C: parental cells; Panels D and E: nonsense shRNA-transfected cells (L-); Panels F and G: TfR1 shRNA stably-transfected cells (L3). * Significantly different from their respective controls (p < 0.05). ^+^Significantly different from the parental cells (p < 0.05).

Considering the high prevalence of iron deficiency in young women and the high tumor recurrence in young breast cancer patients [7,19], association of cellular iron deficiency with VEGF levels and angiogenesis were investigated. Figure 2A shows that background levels of VEGF in the iron deficient TfR1 knockdown L3 cells were approximately 2-fold of those in the parental and nonsense control (L-) cells (bar 4 *vs *1&3). Hypoxia significantly induced VEGF production in both parental and TfR1 knockdown L3 cells. DFO also increased VEGF in the TfR1 knockdown cells, although the difference was not statistically significant. Figure 2B shows that parental, nonsense, and TfR1 knockdown cells all initiated angiogenic responses by recruiting larger and more branched blood vessels resulting in higher vascular density at the injection site (panels B-G) when compared to the non-treated site (panel A). Nonsense control (panels D and E) displayed a greater magnitude of angiogenic responses than parental MDA-MB-231 cells did (panels B and C). Most interestingly, TfR1 knockdown L3-derived xenografts (panels F and G) caused the strongest response among the three treatment groups, indicating that iron-deficient cells possess enhanced angiogenic potential.

### Effects of hypoxia and iron supplementation on LMW iron and ferritin

**Figure 3:**
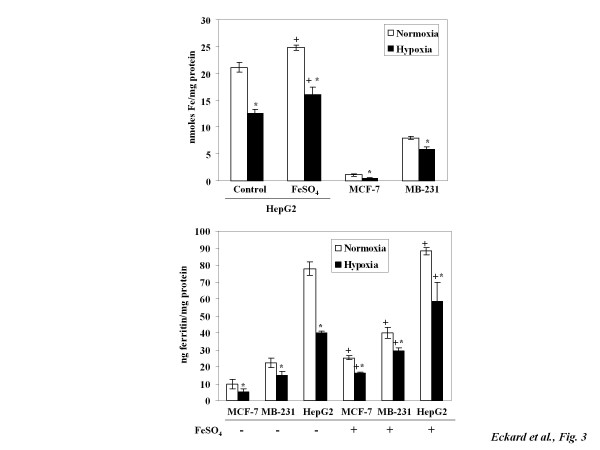
**Hypoxia mediates cellular iron deficiency by decreasing in LMW iron and ferritin levels**. HepG2, MDA-MB-231, and MCF-7 cells were exposed to iron (100 μM FeSO_4_) immediately prior to hypoxia (1% O_2_). After 6 h treatment, LMW iron was determined by calcein assay (A) and ferritin levels by ELISA (B). Data points are means ± SD of four independent experiments (n = 4). * Significantly different from normoxic controls (p < 0.05). ^+ ^Significantly different from respective normoxic or hypoxic controls without iron treatments (p < 0.05).

Anemia is marked by decreased numbers of red blood cells and often results in hypoxia in the breast [20]. To mimic *in vivo *anemic condition, *in vitro *hypoxia at 1% O_2 _was used. Its effects on cellular iron homeostasis were studied in various non-transfected cancer cells, which were more pathophysiologically relevant than the TfR1 knockdown cells. Figure 3A shows that background levels of LMW iron were much higher in the HepG2 cells originated from liver than in the MCF-7 or MDA-MB-231 cells originated from breast. Hypoxia significantly decreased levels of LMW iron in all three types of cells. It is noteworthy that iron given concurrently with hypoxic exposures increased LMW iron levels as compared to the hypoxic controls in HepG2 cells. However, the LMW iron in cells treated with hypoxia and iron was still below those of the control normoxic cells without iron treatment (16.1 ± 1.9 *vs *21.2 ± 0.8 nmol/mg protein).

Figure 3B shows that, similar to the trend observed in LMW iron levels, hypoxia caused a significant decline in ferritin levels in MCF-7, MDA-MB-231, and HepG2 cells. The basal ferritin levels were lower in both MCF-7 and MDA-MB-231 than in HepG2 cells. However, breast cancer cells are more sensitive to iron treatments. For example, levels of ferritin in MCF-7 cells were increased by 120% and 200% under normoxia and hypoxia, respectively. In contrast, ferritin increases in HepG2 cells treated with iron were less than 50% under normoxic and hypoxic conditions.

### Hypoxia increasing levels of TfR1 and IRP binding

**Figure 4:**
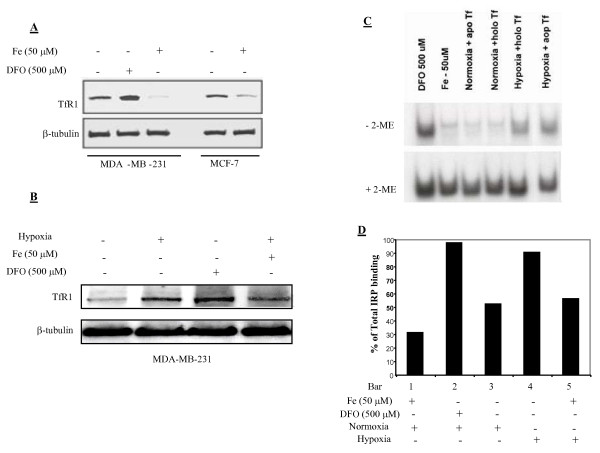
**Hypoxia mediates cellular iron deficiency by increasing TfR1 and IRP binding**. MDA-MB-231 and MCF-7 cells were exposed to Fe (FAC, 50 μM) or DFO (500 μM). Co-treatments were administered 2 h prior to the initiation of hypoxic exposures (37°C, 6 h). Thirty μg total protein/sample were immunoblotted for TfR1 determination (A and B). IRP binding in MDA-MB-231 cells was assessed by EMSA (C). Means of two densitometric analyses were from the IRPs binding experiments (D). Bars represent % IRP binding in samples treated in the absence of ME in comparison to +2-ME controls (n = 2).

Figure 4A shows that iron chelation by DFO increased TfR1 levels in MDA-MB-231 cells, while iron supplementation decreased TfR1 in both MDA-MB-231 and MCF-7 cells. Figure 4B displays that hypoxia increased levels of TfR1 in MDA-MB-231 cells. The addition of iron immediately prior to the hypoxia exposure only slightly, if any, lowered the hypoxia-induced TfR1 increases. Figure 4C shows that DFO greatly increased IRP binding in MDA-MB-231 cells, while iron supplementation decreased it. IRP binding was low under normoxia and its binding was minimally affected by variable Tf saturation rates (either apo-Tf without iron or holo-Tf with 100% iron saturation to its two binding sites) (Figure 4C), suggesting that iron stored in transferrin is not as bioavailable as the inorganic iron in IRP regulation. Figure 4D shows that 6-h hypoxic exposure greatly increased IRP binding, giving results similar to that of iron chelation by DFO. Again, iron supplementation (50 μM, 12-h pretreatment) reduced IRP binding and brought hypoxia-induced IRP binding back to its baseline normoxic levels. Together, these results suggest that hypoxia causes cellular iron deficiency by decreasing LMW iron and ferritin and increasing TfR1 and IRP binding. Iron supplementation can partly negate these hypoxia-induced changes in iron status.

### Effect of cellular iron deficiency on HIF-1α stabilization

**Figure 5:**
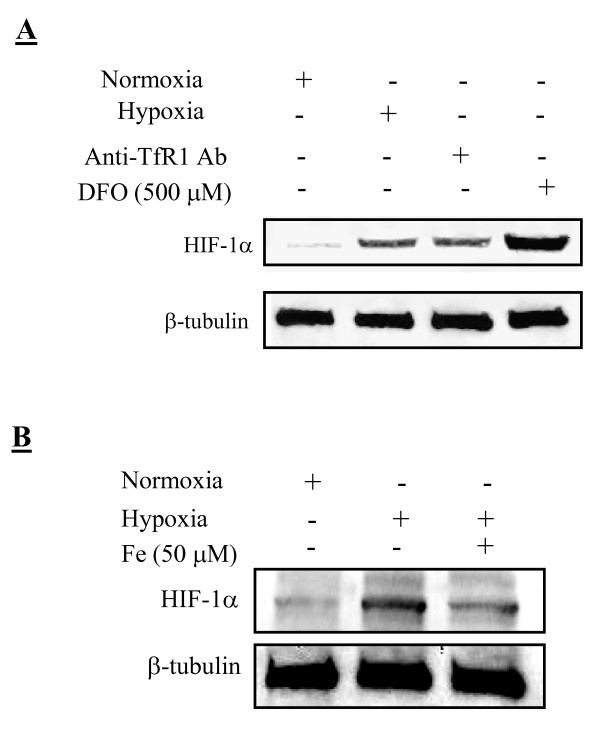
**HIF-1α stabilization in MDA-MB-231 cells by various treatments**. A: normoxia, hypoxia (1% O_2_, 6 h), anti-human TfR1 antibody (Ab, 10 μg/ml, 24 h), or 0.5 mM DFO (6 h). B: normoxia, hypoxia (1% O_2_, 6 h), and hypoxia with Fe pre-treatment (50 μM, 3 h). Samples were analyzed by Western blotting and co-stained for β-tubulin. One representative gel from three independent experiments is shown.

Figure 5A reveals that normoxic MDA-MB-231 cells had a faint staining of HIF-1α, while a 6-h treatment with 0.5 mM DFO under normoxia or a 6-h hypoxia displayed strong HIF-1α stabilization. A 24-h treatment of cells with an anti-TfR1 antibody at a concentration of 10 μg/ml also resulted in HIF-1α stabilization. To test whether iron is capable of altering HIF-1α levels, cells were treated with iron and HIF-1α assessed. Figure 5B shows that iron supplementation under hypoxia leads to HIF-1α destabilization as compared to the hypoxic controls.

### Effects of hypoxia and iron supplements on VEGF and *in vitro *angiogenesis

**Figure 6:**
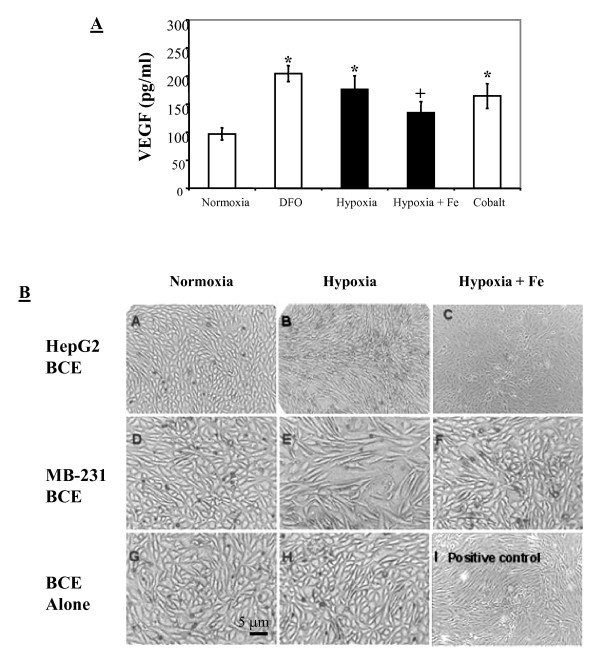
**VEGF levels in culture media of MDA-MB-231 cells and *in vitro *angiogenesis following various treatments**. (A): Tissue culture media from parental MDA-MB-231 cells were collected for VEGF measurements by ELISA; bars represent means + SD of four independent experiments (n = 4). (B): Panels A-C: BCE cells co-cultured with HepG2 cells under normoxia; hypoxia; or hypoxia with 50 μM FAC. Panels D-F: BCE cells co-cultured with MDA-MB-231 cells under normoxia; hypoxia; hypoxia with 50 μM FAC. Panels G-I: BCE cells alone under normoxia; hypoxia; or normoxia with basic fibroblast growth factor (2 ng/ml). * Significantly different from normoxic controls (p < 0.05). ^+^Significantly different from hypoxia alone (p < 0.05).

Figure 6A shows that DFO and hypoxia significantly increased VEGF levels in MDA-MB-231 cells when compared to the controls, as did cobalt, a metal ion used as a positive control for HIF-1α stabilization [21]. Interestingly, iron supplementation caused significant decreases in VEGF levels under hypoxia, suggesting that iron counteracts HIF-1α stabilization and, thus, possibly prevents the processes leading to angiogenesis. To confirm this finding, a co-culture system was used with trans-well membrane inserts containing HepG2 or MDA-MB-231 cells placed in the wells containing BCE cells. As shown in Figure 6B, BCE cells co-cultured with cancer cells under normoxia did not display morphological changes (panels A and D), similar to quiescent BCE cells under normoxia forming a typical cobblestone monolayer on gelatin-coated surfaces (panel G). When BCE cells were cultured alone under hypoxia they appeared quite resilient to hypoxic effects with no visible changes in cell morphology (panel H). However, BCE cells co-cultured with cancer cells under hypoxia showed significant changes in cell morphology (panels B and E). Upon activation, BCE cells became spindle-shaped and arranged in patterns similar to cells treated with basic fibroblast growth factor (panel I). Interestingly, when co-cultures were pretreated with iron under hypoxia, hypoxia-induced BCE activation was greatly diminished (panels C and F). The morphology of the iron- and hypoxia-treated BCE cells remained similar to that of the cells under normoxic co-culture conditions.

## Discussion

Estrogen and family history are two of the most significant and well-characterized risk factors for breast cancer. However, family history and estrogen cannot fully explain the higher breast cancer recurrence rates and greater aggressiveness with mostly triple negative breast tumors in young, pre-menopausal women as compared to the older post-menopausal women [22-24]. Because of high prevalence of iron deficiency in young women [7], we have previously hypothesized that iron deficiency may play an important role in the poor outcome of young breast cancer patients [18]. Here we provide evidence that cellular iron deficiency contributes to HIF-1α stabilization, VEGF formation, and angiogenesis, all of which are important in carcinogenesis, metastasis, and breast cancer recurrence [25].

To date, most research involving iron in cancer has been focused on iron overload causing oxidant-mediated cancer promotion [26]. Our contention that iron deficiency plays a role in breast cancer recurrence, appears contradictory to the findings that iron overload contributes to cancer [26]. However, there are important differences that separate the two lines of investigations and their conclusions. First, the aforementioned studies examined the role of iron overload in cancer initiation and promotion stages, for example, by fortifying diet with iron in the presence or absence of a carcinogen [27-29]. Second, epidemiological studies have examined the iron status of the whole body attempting to link increased iron with increased risk of cancer development [26,30]. The present study focused on the other end of the iron spectrum and examined how cellular iron deficiency in cancer cells could affect signal transduction pathways leading to tumor angiogenesis, a key step necessary for cancer metastasis and its recurrence.

It is well known that when a new vasculature forms and penetrates into the tumor core, primary tumor cells can enter into the circulation and form secondary tumor in distant organs. Once the tumors recur, the disease becomes incurable resulting in a high death rate. To test our hypothesis [18], triple negative MDA-MB-231 cells were stably-transfected with TfR1 shRNA and used as iron deficient cancer cells. This mild TfR1 knockdown was not lethal and provided the most appropriate conditions to prove our hypothesis because: (a) cellular iron homeostasis is mostly maintained by TfR1 (iron uptake) and ferritin (iron storage) [11,31,32], and unaltered cells can readily adapt to iron level changes in the environment using endogenous IRPs, which can alter mRNA stability, transcription, and translation of TfR1 and ferritin; (b) cells "primed" under iron deficient *in vitro *culture conditions or even 3 D tissue culture can reach iron equilibrium in the *in vivo *environment once these cells are injected into the animals [33]. In this study, we found that stable TfR1 knockdown cells were constitutively iron-deficient as revealed by the decreased levels of ferritin, a hallmarker of iron status (Figure 1C).

HIF-1α and VEGF were used as the endpoints of the present study because either or both can elicit an array of effects, from initiating angiogenesis to tumor metastasis, [17,34]. Overexpression of VEGF by MCF-7 cells was shown to promote estrogen-independent tumor growth *in vivo *[35]. Patients with early stage breast cancer who have tumors with elevated levels of VEGF were reported to have a higher likelihood of recurrence or death than patients having tumors with a low angiogenic potential [25]. VEGF and VEGF receptors have proven effective targets for current oncology therapies. However, whether iron deficiency is related to VEGF and angiogenesis is unclear, and if yes, molecular mechanism remains unknown. Here, we showed that iron deficient cells by knocking down TfR1 produced more VEGF and induced higher angiogenesis in nude mice than parental and nonsense control cells (Figure 2). Our results are in agreement with the previous study showing that TfR1 antibody, which specifically blocks the binding region of TfR1 for transferrin, inhibited breast cancer cell proliferation [36] and increased VEGF formation [37]. Additionally, we showed that iron chelation by DFO or blocking iron uptake through TfR1 antibody stabilizes HIF-1α under normoxic conditions (Figure 5). These results stress the importance of iron deficiency in the process of HIF-1α stabilization, possibly by inhibiting HIF degrading prolyl-4-hydroxylases [38,39]. Whether these iron deficient cells with higher potentials of angiogenesis are more capable of metastasizing into other organs await further investigations.

Anemia is functionally defined as having inadequate numbers of red blood cells to maintain tissue oxygenation. It has been shown that hypoxia is more intensified in young and anemic breast cancer patients [20]. This led us to investigate how hypoxia affects cellular iron homeostasis. Our results showed that hypoxia decreases levels of LMW iron in HepG2, MCF-7, and MDA-MB-231 cells (Figure 3). This suggests a strong iron demand during hypoxic exposures [40]. Although the overall trends of hypoxia-mediated LMW iron decreases were the same, HepG2 cells derived from human hepatocarcinoma had significantly higher LMW iron content than breast cancer cells. These differences may stem from differences in iron metabolism profiles between the originating tissues of the cell lines. Hepatocytes are known for having high iron content because of the liver's role in clearing and processing aged red blood cells. Interestingly, breast cancer cells were much more responsive to iron supplementation than HepG2 cells were, as shown by changes in levels of ferritin (Figure 4B).

To confirm that the observed changes in ferritin and TfR1 are a result of hypoxia-mediated iron decrease, IRP binding to IRE was determined (Figure 4). Our results showed that IRP binding to IRE increased during hypoxia, while iron supplementation decreased IRP binding. The IRP binding patterns in these two experiments suggest that decreases in iron levels during hypoxia caused a shift in iron metabolism to a state favoring iron uptake and signaling cellular iron deficiency [41,42]. Our results, along with the established findings of HIF-mediated increase in TfR1 expression [43], indicate that IDA induces hypoxia and stabilizes HIF-1α and, in turn, hypoxia increases cellular iron uptake to combat iron deficiency through negative feedback of the hypoxic conditions [44].

To further associate iron deficiency with HIF-1α, VEGF, and angiogenesis, we showed that iron supplementation caused a destabilization of HIF-1α (Figure 5B), a result which supports the idea that iron deficiency contributes to HIF-1α induction and stabilization. The effects of iron supplementation also extended beyond cellular HIF stabilization and into downstream HIF signaling, which lead to a lower level of VEGF and a decline in the *in vitro *angiogenesis (Figure 6).

## Conclusions

Our study is distinct from previous studies on iron overload and its contribution to cancer initiation and promotion *via *oxidative stress pathways [26]. Our study has demonstrated an important role for cellular iron deficiency in HIF-1α stabilization, VEGF formation, and angiogenesis, suggesting that systemic iron deficiency and anemia in young breast cancer patients may make them more susceptible to tumor recurrence through the same mechanism.

## Methods

### Chemical reagents

DFO, ferric ammonium citrate (FAC), ferrous sulfate heptahydrate (FeSO_4_•7 H_2_O), β-mercaptoethanol (ME), agar, and DNA ligase kit LIG-1 were purchased from Sigma (St. Louis, MO). Other reagents were: SDS-Tris-HCl or non-denaturing polyacrylamide gels, nitrocellulose membranes, (Bio-Rad Laboratories, Hercules, CA); M-PER mammalian protein extraction reagent, calcein (Molecular Probes); mini-Prep kit for vector DNA isolation (Qiagen); anti-human TfR1 antibody (Research Diagnostics, Flanders, NJ); Lipofectamine™ 2000 (Invitrogen, Carlsbad, CA); anti-HIF-1α antibody (BD Biosciences, Palo Alto, CA); shRNA vector sequences, pSilencer expression vector kit, RNA isolation kits, DNA-free and RNAqueous (Ambion, Inc., Austin, TX).

### Cell culture

Human triple negative breast cancer cell line MDA-MB-231 was used for shRNA knockdown. For comparison, human breast cancer ER^+ ^MCF-7 cells and human hepatocarcinoma HepG2 cells with high background levels of intracellular iron were also tested (ATCC, Manassas, VA). Bovine capillary endothelial (BCE) cells, a kind gift from Dr. Paolo Mignatti, were used for *in vitro *angiogenesis (Department of Cell Biology, NYU School of Medicine). Growth of each cell line was initiated with the recommended culture conditions from the supplier. All cell lines were then adapted to alpha-minimal essential medium (α-MEM) supplemented with 5% FBS, 2 mM L-glutamine, and 1X antibiotic/antimycotic solution. This measure was taken to prevent confounding outcomes resulting from changing medium during co-culture experiments. α-MEM was the main culture medium because α-MEM is essentially iron-free, thus providing maximal control of the iron levels (by supplementation) in the culture environment.

Unless otherwise noted, the initial growth medium was removed 18-24 h prior to experiments and replaced with the iron-free α-MEM containing serum-replacement supplement at 5 μg/l selenium, 10 mg/l insulin, 5.5 mg/l transferrin, and 2 mg/l ethanolamine. These additives allowed cells to be maintained and even propagated without serum for periods ranging from 72 h to over a week. Cells were maintained in a humidified tissue culture incubator at 37°C under 5% CO_2_. Normoxia exposures were carried out in the same incubator, while hypoxic exposures were performed in a specially designed exposure system with hypoxic gas (1% O_2_, 5% CO_2_, and N_2 _balance).

### TfR1 knockdown in MDA-MB-231 cells by shRNA

Three sequences of shRNA duplexes for human TfR1 as well as control shRNA were purchased from Ambion Inc. (Austin, TX). The TfR1 shRNA oligonucleotide sequences were as follows:

L1: 5'-GATCCGGCCTTAATATGTTAACCTTTCAAGAGAAGGTTAACATATTAAGGCCTTA-3'

L2: 5'-GATCCGGCCAATGTCACAAAACCATTCAAGAGATGGTTTTGTGACATTGGCCTT A-3'

L3: 5'-GATCCGGTGTAGTGGAAGTATCTGTTCAAGAGACAGATACTTCCACTACACCTT A-3'

After ligating into a *pSilencer *4.1-CMV neo vector, MDA-MB-231 cells were seeded into 24-well plates and cultured until about 50% confluent, then transfected with 80 pmol TfR1 shRNA in Lipofectamine™ 2000 for 24 h according to the manufacturer's instructions. Cells treated with Lipofectamine™ 2000 alone and control non-silencing shRNA were used as mock-transfected controls. After 24 h, transiently-transfected cells were used for some biochemical measurements and the remaining cells were transferred to 25-cm^2 ^flasks for selection. Stably-transfected TfR1 knockdown cells (L1-L3) as well as nonsense control cells (L-) were selected with neomycin at 2 mg/ml [45]. To verify TfR1 suppression, transfected cells were scrapped, homogenized, and analyzed by Western blot.

### *In vivo *angiogenesis using TfR1 shRNA knockdown cells

Three groups, each consisting of four 6-week old female nude mice (Jackson Labs, Bar Harbor, ME) were treated as follows: parental MDA-MB-231, MDA-MB-231 cells stably-transfected with nonsense shRNA vector (L-), and MDA-MB-231 cells stably-transfected with TfR1 shRNA vector (L3). After harvesting, 2.5 × 10^6 ^cells were suspended in 0.2 ml of 1:1 (vol:vol) mixture of PBS and the same batch of Matrigel™. Cells were inoculated subcutaneously (0.2 ml) into both flanks of the nude mice. Seventy-two hours post-inoculation, the subcutaneous Matrigel plugs and skin surrounding the injection site were excised, trans-illuminated and photographed.

### Iron regulatory proteins (IRPs) electro-mobility shift assay (EMSA)

The binding activities of IRPs to IRE were examined by EMSA [46,47]. Following treatment, MDA-MB-231 cells were lysed in a lysis buffer containing 10 mM Hepes (pH 7.5), 3 mM MgCl_2_, 40 mM KCl, 5% glycerol, 0.3% NP-40, and a complete protease inhibitor cocktail. The ^32^P-labeled IRE probe was synthesized by *in vitro *transcription using the linearized pSPT-fer plasmid as the template (a kind gift from Dr. L.C. Kühn, ISREC, Switzerland), which contains human ferritin H-chain IRE. After dividing samples into two equal parts, 2-mercaptoethanol (2-ME) was added to one part of the sample at a 2% final concentration. The addition of 2-ME creates a reducing environment, which facilitates maximal binding of all present IRP proteins, and was used as the internal control for IRP binding normalization. The two samples (+/- 2-ME; 2 μg proteins each) were incubated at room temperature with an excess amount of ^32^P-labeled IRE probe (4 × 10^4 ^cpm) in 20 μl reaction buffer (10 mM Hepes, pH 7.5, 3 mM MgCl_2_, 40 mM KCl, 5% glycerol, 0.07% NP-40, and a complete protease inhibitor cocktail). After 20 min incubation, 1 unit RNase and 100 μg heparin were added, and the mixture was incubated for an additional 10 min period. The reaction mixture was separated on a 5% non-denaturing polyacrylamide gel. The gel was dried and the amount of radioactive material assessed by exposure to X-ray film. The IRP binding was calculated by comparing the band density of the sample without 2-ME to the sample with 2-ME.

### Analysis of low molecular weight (LMW) iron

Levels of LMW iron in cells were determined using an iron-sensitive fluorescent calcein as previously described [48]. Briefly, calcein binds iron stoichiometrically, with its fluorescence quenched upon calcein-Fe complex formation. By adding DFO, the fluorescence specifically quenched by iron can be regenerated. Therefore, the difference between fluorescence readings of the same sample with and without DFO is directly proportional to the amounts of LMW iron. The levels of LMW iron are expressed as nmol iron/mg protein.

### Determination of TfR1 and ferritin

TfR1 levels were measured by Western blot as follows: cell lysates (30 μg protein) were subjected to 12% SDS-polyacrylamide gel electrophoresis. After transferring onto nitrocellulose membranes, the membranes were blocked with 5% non-fat dry milk in Tris-buffered saline containing 0.05% Tween 20, probed together with antibodies against TfR1 (1:500) and β-tubulin (1:3000), and then visualized with peroxidase-conjugated anti-mouse antibody using Western Lightning Plus Chemiluminescence Reagent (Perkin-Elmer). Levels of ferritin in cell lysates were determined by enzyme-linked immunosorbent assay (ELISA) according to the method previously developed in our laboratory [49]. Total protein in the cell lysates was determined using bicinchoninic acid and the results expressed as ng ferritin/mg protein.

### Measurements of VEGF

Levels of VEGF in the tissue culture media were measured using pre-coated kits (Biosource Inc., Camarillo, CA) following the Manufacturer's instruction. Data were expressed as pg VEGF/ml culture media.

### *In vitro *angiogenesis using co-cultures of cancer cells and BCE

Cells were initially cultured separately in serum-containing growth medium; cancer cells in 24-well culture inserts containing a 0.4 μm porous membrane, and BCE cells in a 24-well, gelatin-coated plate. After cells were grown to 75% confluence, the medium was replaced with serum-free medium, and the cells were grown for an additional 24 h. To initiate the co-culture, inserts containing cancer cells were placed into wells containing BCE cells. The porous membrane kept cells physically separated while allowing secreted factors to pass through the membrane and be mixed with the medium covering the BCE cells. Prior to the co-culture, photographs of BCE cells were taken for a reference. Twenty four h after exposures to the factors generated by cancer cells grown on the inserts, BCE cells were photographed again.

### Statistical analysis

The experimental differences were determined by two-tailed Student's *t*-test. Graphed data represent the means ± SD of at least three experiments except that the IRP binding assays were done twice. A confidence level of p < 0.05 was taken to represent a significant difference in all cases.

## Abbreviations

2-ME: 2-mercaptoethanol; BCE: bovine capillary endothelial cells; DFO: deferoxamine; EMSA: electro-mobility shift assay; ER: estrogen receptor; FAC: ferric ammonium citrate; HER2: human epidermal growth factor receptor 2; HIF-1α: hypoxia inducible factor-1 alpha; HPO: hypothalamic-pituitary-ovarian; IDA: iron deficiency anemia; IRE: iron responsive element; IRP: iron regulatory protein; LMW: low molecular weight; PR: progesterone receptor; TF: transferrin; TFR1: transferrin receptor; SHRNA: small interfering RNA; VEGF: vascular endothelial growth factor.

## Competing interests

The authors declare that they have no competing interests.

## Authors' contributions

JE carried out shRNA knockdown and the *in vivo *and *in vitro *angiogenesis and helped to draft the manuscript; JD, JJ, and QY evaluated cellular iron status in all three types of cells, JW and HC performed the IRP binding assay, MC and KF participated in the design of the study and helped to draft the manuscript, XH conceived of the study and drafted the manuscript. All authors read and approved the final manuscript.

## References

[B1] Casalini P Carcangiu ML Tammi R Auvinen P Kosma VM Valagussa P Greco M Balsari A Menard S Tagliabue E Two distinct local relapse subtypes in invasive breast cancer: effect on their prognostic impact Clin Cancer Res 2008 14 25 31 10.1158/1078-0432.CCR-07-0450 18172248

[B2] Klauber-DeMore N Tumor biology of breast cancer in young women Breast Dis 2005 23 9 15 16823162 10.3233/bd-2006-23103

[B3] Jemal A Ward E Thun MJ Recent trends in breast cancer incidence rates by age and tumor characteristics among U.S. women Breast Cancer Res 2007 9 R28 10.1186/bcr1672 17477859 PMC1929089

[B4] Ali MA Akhmedkhanov A Zeleniuch-Jaquotte A Toniolo P Frenkel K Huang X Reliability of serum iron, ferritin, nitrite, and association with risk of renal cancer in women Cancer Det Prev 2003 27 116 121 10.1016/S0361-090X(03)00027-8 PMC2965440 12670522

[B5] Atwood CS Meethal SV Liu T Wilson AC Gallego M Smith MA Bowen RL Dysregulation of the hypothalamic-pituitary-gonadal axis with menopause and andropause promotes neurodegenerative senescence J Neuropathol Exp Neurol 2005 64 93 103 15751223 10.1093/jnen/64.2.93

[B6] Chakraborty TR Gore AC Aging-related changes in ovarian hormones, their receptors, and neuroendocrine function Exp Biol Med (Maywood) 2004 229 977 987 15522833 10.1177/153537020422901001

[B7] Zimmermann MB Hurrell RF Nutritional iron deficiency Lancet 2007 370 511 520 10.1016/S0140-6736(07)61235-5 17693180

[B8] Cavill I Auerbach M Bailie GR Barrett-Lee P Beguin Y Kaltwasser P Littlewood T Macdougall IC Wilson K Iron and the anaemia of chronic disease: a review and strategic recommendations Curr Med Res Opin 2006 22 731 737 10.1185/030079906X100096 16684434

[B9] Hercberg S Preziosi P Galan P Iron deficiency in Europe Public Health Nutr 2001 4 537 545 10.1079/PHN2001139 11683548

[B10] Weiss G Gordeuk VR Benefits and risks of iron therapy for chronic anaemias Eur J Clin Invest 2005 35 Suppl 3 36 45 10.1111/j.1365-2362.2005.01529.x 16281957

[B11] Pantopoulos K Iron metabolism and the IRE/IRP regulatory system: an update Ann N Y Acad Sci 2004 1012 1 13 10.1196/annals.1306.001 15105251

[B12] Rouault TA The role of iron regulatory proteins in mammalian iron homeostasis and disease Nat Chem Biol 2006 2 406 414 10.1038/nchembio807 16850017

[B13] Muckenthaler MU Galy B Hentze MW Systemic Iron Homeostasis and the Iron-Responsive Element/Iron-Regulatory Protein (IRE/IRP) Regulatory Network Annu Rev Nutr 2008 28 197 213 10.1146/annurev.nutr.28.061807.155521 18489257

[B14] Recalcati S Minotti G Cairo G Iron regulatory proteins: from molecular mechanisms to drug development Antioxid Redox Signal 2010 in press 20214491 10.1089/ars.2009.2983

[B15] Dongiovanni P Valenti L Ludovica Fracanzani A Gatti S Cairo G Fargion S Iron depletion by deferoxamine up-regulates glucose uptake and insulin signaling in hepatoma cells and in rat liver Am J Pathol 2008 172 738 747 10.2353/ajpath.2008.070097 18245813 PMC2258266

[B16] Salnikow K Donald SP Bruick RK Zhitkovich A Phang JM Kasprzak KS Depletion of intracellular ascorbate by the carcinogenic metals nickel and cobalt results in the induction of hypoxic stress J Biol Chem 2004 279 40337 40344 10.1074/jbc.M403057200 15271983

[B17] Bertout JA Patel SA Simon MC The impact of O2 availability on human cancer Nat Rev Cancer 2008 8 967 975 10.1038/nrc2540 18987634 PMC3140692

[B18] Huang X Does iron have a role in breast cancer? Lancet Oncol 2008 9 803 807 10.1016/S1470-2045(08)70200-6 18672216 PMC2577284

[B19] Han W Kim SW Park IA Kang D Kim SW Youn YK Oh SK Choe KJ Noh DY Young age: an independent risk factor for disease-free survival in women with operable breast cancer BMC Cancer 2004 4 82 10.1186/1471-2407-4-82 15546499 PMC545947

[B20] Vaupel P Mayer A Briest S Hockel M Hypoxia in breast cancer: role of blood flow, oxygen diffusion distances, and anemia in the development of oxygen depletion Adv Exp Med Biol 2005 566 333 342 full_text 16594170 10.1007/0-387-26206-7_44

[B21] Kang GS Li Q Chen H Costa M Effect of metal ions on HIF-1alpha and Fe homeostasis in human A549 cells Mutat Res 2006 610 48 55 16877034 10.1016/j.mrgentox.2006.06.012

[B22] Cavalieri E Frenkel K Liehr JG Rogan E Roy D Estrogens as endogenous genotoxic agents--DNA adducts and mutations J Natl Cancer Inst Monogr 2000 75 93 10963621 10.1093/oxfordjournals.jncimonographs.a024247

[B23] Rennert G Bisland-Naggan S Barnett-Griness O Bar-Joseph N Zhang S Rennert HS Narod SA Clinical outcomes of breast cancer in carriers of BRCA1 and BRCA2 mutations N Engl J Med 2007 357 115 123 10.1056/NEJMoa070608 17625123

[B24] Russo J Hasan Lareef M Balogh G Guo S Russo IH Estrogen and its metabolites are carcinogenic agents in human breast epithelial cells J Steroid Biochem Mol Biol 2003 87 1 25 10.1016/S0960-0760(03)00390-X 14630087

[B25] Gasparini G Prognostic value of vascular endothelial growth factor in breast cancer Oncologist 2000 5 Suppl 1 37 44 10.1634/theoncologist.5-suppl_1-37 10804090

[B26] Huang X Iron overload and its association with cancer risk in humans: evidence for iron as a carcinogenic metal Mutat Res 2003 533 153 171 14643418 10.1016/j.mrfmmm.2003.08.023

[B27] Hardman WE Barnes CJ Knight CW Cameron IL Effects of iron supplementation and ET-18-OCH3 on MDA-MB 231 breast carcinomas in nude mice consuming a fish oil diet Br J Cancer 1997 76 347 354 9252202 10.1038/bjc.1997.389 PMC2224052

[B28] Ilsley JN Leung SF Belinsky GS Guda K Zhang Q Huang X Blumberg JB Milbury PE Roberts Ii LJ Stevens RG Rosenberg DW Dietary iron promotes azoxymethane-induced colon tumors in mice Nutr Cancer 2004 49 162 169 10.1207/s15327914nc4902_7 15489209

[B29] Thompson HJ Kennedy K Witt M Juzefyk J Effect of dietary iron deficiency or excess on the induction of mammary carcinogenesis by 1-methyl-1-nitrosourea Carcinogenesis 1991 12 111 114 10.1093/carcin/12.1.111 1988169

[B30] Weinberg ED Iron in neoplastic disease Nutr Cancer 1983 4 223 233 10.1080/01635588209513761 6302639

[B31] Andrews NC Schmidt PJ Iron homeostasis Annu Rev Physiol 2007 69 69 85 10.1146/annurev.physiol.69.031905.164337 17014365

[B32] Ganz T Nemeth E Regulation of iron acquisition and iron distribution in mammals Biochim Biophys Acta 2006 1763 690 699 10.1016/j.bbamcr.2006.03.014 16790283

[B33] Lee GY Kenny PA Lee EH Bissell MJ Three-dimensional culture models of normal and malignant breast epithelial cells Nat Methods 2007 4 359 365 10.1038/nmeth1015 17396127 PMC2933182

[B34] Semenza GL Hypoxia-inducible factor 1 and cancer pathogenesis IUBMB Life 2008 60 591 597 10.1002/iub.93 18506846

[B35] Guo P Fang Q Tao HQ Schafer CA Fenton BM Ding I Hu B Cheng SY Overexpression of vascular endothelial growth factor by MCF-7 breast cancer cells promotes estrogen-independent tumor growth in vivo Cancer Res 2003 63 4684 4691 12907650

[B36] Jiang XP Elliott RL Head JF Manipulation of iron transporter genes results in the suppression of human and mouse mammary adenocarcinomas Anticancer Res 2010 30 759 765 20392994

[B37] Jones DT Trowbridge IS Harris AL Effects of transferrin receptor blockade on cancer cell proliferation and hypoxia-inducible factor function and their differential regulation by ascorbate Cancer Res 2006 66 2749 2756 10.1158/0008-5472.CAN-05-3857 16510596

[B38] Gerald D Berra E Frapart YM Chan DA Giaccia AJ Mansuy D Pouyssegur J Yaniv M Mechta-Grigoriou F JunD reduces tumor angiogenesis by protecting cells from oxidative stress Cell 2004 118 781 794 10.1016/j.cell.2004.08.025 15369676

[B39] Semenza GL Hypoxia-inducible factor 1 (HIF-1) pathway Sci STKE 2007 2007 cm8 10.1126/stke.4072007cm8 17925579

[B40] Robach P Cairo G Gelfi C Bernuzzi F Pilegaard H Vigano A Santambrogio P Cerretelli P Calbet JA Moutereau S Lundby C Strong iron demand during hypoxia-induced erythropoiesis is associated with down-regulation of iron-related proteins and myoglobin in human skeletal muscle Blood 2007 109 4724 4731 10.1182/blood-2006-08-040006 17311997

[B41] Peyssonnaux C Zinkernagel AS Schuepbach RA Rankin E Vaulont S Haase VH Nizet V Johnson RS Regulation of iron homeostasis by the hypoxia-inducible transcription factors (HIFs) J Clin Invest 2007 117 1926 1932 10.1172/JCI31370 17557118 PMC1884690

[B42] Sanchez M Galy B Muckenthaler MU Hentze MW Iron-regulatory proteins limit hypoxia-inducible factor-2alpha expression in iron deficiency Nat Struct Mol Biol 2007 14 420 426 10.1038/nsmb1222 17417656

[B43] Semenza GL Hypoxia-inducible factor 1: oxygen homeostasis and disease pathophysiology Trends Mol Med 2001 7 345 350 10.1016/S1471-4914(01)02090-1 11516994

[B44] Peyssonnaux C Nizet V Johnson RS Role of the hypoxia inducible factors HIF in iron metabolism Cell Cycle 2008 7 28 32 18212530 10.4161/cc.7.1.5145

[B45] Chen CL Hsieh YT Chen HC Phosphorylation of adducin by protein kinase Cdelta promotes cell motility J Cell Sci 2007 120 1157 1167 10.1242/jcs.03408 17341583

[B46] Rothenberger S Mullner EW Kuhn LC The mRNA-binding protein which controls ferritin and transferrin receptor expression is conserved during evolution Nucleic Acids Res 1990 18 1175 1179 10.1093/nar/18.5.1175 2157191 PMC330432

[B47] Wu J Eckard J Chen H Costa M Frenkel K Huang X Altered iron homeostasis involvement in arsenite-mediated cell transformation Free Radic Biol Med 2006 40 444 452 10.1016/j.freeradbiomed.2005.08.035 16443159 PMC2955321

[B48] Ali A Zhang Q Dai J Huang X Calcein as a fluorescent iron chemosensor for the determination of low molecular weight iron in biological fluids Biometals 2003 16 285 293 10.1023/A:1020642808437 12572687

[B49] Zhang Q Huang X Induction of ferritin and lipid peroxidation by coal samples with different prevalence of coal workers' pneumoconiosis: role of iron in the coals Am J Ind Med 2002 42 171 179 10.1002/ajim.10101 12210686

